# Roles of macrophages in lupus nephritis

**DOI:** 10.3389/fphar.2024.1477708

**Published:** 2024-11-14

**Authors:** Yaqian Cheng, Lulu Liu, Yufei Ye, Yingxue He, Wenwen Hu, Haiyan Ke, Zhi-Yong Guo, Guojian Shao

**Affiliations:** ^1^ Department of Nephrology, Wenzhou Central Hospital, Wenzhou, China; ^2^ Department of Nephrology, Shanghai Changhai Hospital, Naval Medical University, Shanghai, China

**Keywords:** M1 macrophages, M2 macrophages, SLE, lupus nephritis, LN

## Abstract

LN is a serious complication of systemic lupus erythematosus (SLE), affecting up to 60% of patients with SLE and may lead to end-stage renal disease (ESRD). Macrophages play multifaceted roles in the pathogenesis of LN, including clearance of immune complexes, antigen presentation, regulation of inflammation, and tissue repair. Macrophages are abundant in the glomeruli and tubulointerstitium of LN patients and are positively correlated with serum creatinine levels and the severity of renal pathology. It has been shown that the infiltration of macrophages is closely associated with several clinical indicators, such as serum creatinine and complement C3 levels, anti-dsDNA antibody titers, Austin score, interstitial fibrosis and renal tubular atrophy. Moreover, cytokines expressed by macrophages were upregulated at LN onset and downregulated after remission, suggesting that macrophages may serve as markers of LN pathogenesis and remission. Therapies targeting macrophages have been shown to alleviate LN. There are two main types of macrophages in the kidney: kidney-resident macrophages (KRMs) and monocyte-derived macrophages (MDMs). KRMs and MDMs play different pathological roles in LN, with KRMs promoting leukocyte recruitment at sites of inflammation by expressing monocyte chemokines, while MDMs may exacerbate autoimmune responses by presenting immune complex antigens. Macrophages exhibit high plasticity and can differentiate into various phenotypes in response to distinct environmental stimuli. M1 (proinflammatory) macrophages are linked to the progression of active SLE, whereas the M2 (anti-inflammatory) phenotype is observed during the remission phase of LN. The polarization of macrophages in LN can be manipulated through multiple pathways, such as the modulation of signaling cascades including TLR 2/1, S1P, ERS, metabolic reprogramming, and HMGB1. This paper provides a comprehensive overview of the role of macrophages in the progression of lupus nephritis (LN), and elucidates how these cells and their secretory products function as indicators and therapeutic targets for the disease in the context of diagnosis and treatment of LN.

## 1 Introduction

Systemic lupus erythematosus (SLE) is an autoimmune disease that affects various organs, causing a series of complications. Lupus nephritis (LN) is one of the most serious complications of SLE, occurring in up to 60% of SLE patients (Chan et al., 2023). The prognosis of LN is generally poor, with approximately 7.9% of patients progressing to end-stage renal disease (ESRD) 16.5 years after the onset of LN (Chan et al., 2023), making LN one of the leading causes of death in SLE patients (Wang et al., 2015; Tselios et al., 2019). LN is initiated by the defective clearance of apoptotic cells, as well as the loss of tolerance to autoantigens (Liu and Davidson, 2012; Mistry and Kaplan, 2017). These events lead to abnormal activation of the immune system, which triggers a series of inflammatory responses resulting in renal damage. Macrophages undertake a multifaceted role in the etiology of LN, engaging not merely in the elimination of immune complexes and antigen presentation, but also modulating inflammation and tissue repair via diverse biological pathways. On the one hand, activated macrophages and other antigen-presenting cells phagocytose cellular fragments and present antigens to T and B cells, resulting in the production of antibodies. In combination with autoantibodies and autoantigens, immune complexes are deposited in the kidneys and activate Fc receptors and complement systems, leading to kidney damage (Flores-Mendoza et al., 2018). On the other hand, macrophages, as pivotal players in the innate immune system, possess surface Toll-like receptors (TLRs) that, upon recognizing self-nucleic acids and other autoantigens, possess the potential to exacerbate autoimmune responses (Lind et al., 2022). Macrophages also play a pivotal role in activating and regulating the complement system, thereby significantly influencing the progression of LN (Richoz et al., 2022; Arazi et al., 2019). Macrophages possess the capability to modulate the functionality of neutrophils, encompassing the generation of NETs (neutrophil extracellular traps) and the regulation of LDGs (leukocyte-derived granules), subsequently influencing the inflammatory cascade within lymph nodes (Manda-Handzlik et al., 2023). Furthermore, once activated, macrophages are capable of secreting a diverse series of cytokines, including IL-6, TNF-α, and IL-1β, which subsequently stimulate inflammatory activities within kidneys. As the inflammatory response reaches an advanced stage, macrophages undergo polarization into a reparative phenotype, actively participating in the restoration and repair process of kidney tissue (Nakatani et al., 2010; Kim et al., 2020a). Thus, it is apparent that macrophages are involved in the pathology of LN in many ways.

In this article, we summarize the interactions between macrophages and intrinsic renal cells in LN, the feasibility of the use of macrophages as biomarkers for LN, and the current status of targeting macrophages for the treatment of LN, which may facilitate a better understanding of the roles of macrophages in LN and perhaps provide a reference for further understanding the pathogenesis of LN and the development of related interventions.

## 2 Macrophages in LN

Macrophages are members of the innate immune system that are crucial for inflammatory responses and host defense. They are mainly responsible for recognizing, phagocytosing, and degrading invading pathogens and cellular fragments and then presenting the processed antigenic components to other immune cells to drive the immune response (Bajgar and Krejčová, 2023; Muntjewerff et al., 2020). Furthermore, during the early stage of inflammation, macrophages also amplify the inflammatory response by releasing a series of cytokines and chemokines that facilitate the recruitment of their effector cells. In addition to promoting inflammation, macrophages play important roles in tissue repair and fibrosis (Bell and Conway, 2022).

Macrophages are abundant in the glomeruli and tubular interstitium of LN patients (Hu and Chen, 2022; Sahu et al., 2014; Sung et al., 2017). The number of infiltrating macrophages is positively correlated with the level of serum creatinine and the severity of renal pathology (Chen J. et al., 2022). Olmes et al. (2016) analyzed the infiltration of macrophages in the kidneys of patients with type II-V lupus nephritis (classified according to the ISN/RPS system) and reported that the number of macrophages was closely related to several clinical indicators, such as the levels of serum creatinine and complement C3, the anti-dsDNA antibody titer, the Austin score, interstitial fibrosis and tubular atrophy. Moreover, Schiffer et al. (2008) reported that cytokines expressed by activated macrophages are upregulated at proteinuria onset and downregulated after remission induction in NZB/NZW F1 mice, suggesting that activated macrophages may serve as markers of disease onset and remission in LN. In addition, treatments targeting macrophages have been shown to alleviate LN (Chalmers et al., 2015; Chalmers et al., 2017; Zhang et al., 2019). Thus, we can deduce that macrophages play an important role in the process of LN.

There are two types of macrophages in the kidney: kidney-resident macrophages (KRMs) and monocyte-derived macrophages (MDMs). We can distinguish between them by the surface markers, as the former highly expresses F4/80 while the latter highly expresses CD11b (Cheung et al., 2022). KRMs are derived from several different sources, including the yolk sac, fetal liver, and bone marrow (Schulz et al., 2012; Ginhoux and Guilliams, 2016). The first wave of kidney macrophages is derived from erythroid myeloid progenitors (EMPs) in the yolk sac in mice. These EMPs differentiate into premacrophages without monocytic intermediates (Munro and Hughes, 2017). Research in a fate-mapping mouse model revealed that the proportion of yolk sac-derived macrophages in the kidney rapidly decreases beginning at embryonic day 13.5 and is only 2%–3% at birth (Hoeffel et al., 2015). However, fetal monocytes gradually populate the mouse kidney beginning at embryonic day 13.5 (Hoeffel et al., 2015). During this stage, EMPs originating from the yolk sac enter the fetal liver and proliferate rapidly, where they differentiate into monocyte intermediates or premacrophages, which leave the liver to reside in tissues and become KRMs (Hoeffel et al., 2015; Epelman et al., 2014). Notably, fetal monocytes but not yolk sac-derived macrophages give rise to the vast majority of KRMs in the adult kidney (Liu et al., 2020). In addition, bone marrow-derived peripheral monocytes differentiate into macrophages to supplement the KRM pool within a certain limit throughout adulthood (Liu et al., 2020; Dick et al., 2022). Supported by a large amount of evidence, we are familiar with the development of KRMs in mice, yet little is known about this topic in humans. Recently, Bian et al. (2020) analyzed hematopoietic cells from human embryos via single-cell sequencing and reported that early embryonic macrophages in humans exhibit a developmental process similar to that of macrophages in mice.

Comparatively, the source of MDMs is well known. Hematopoietic stem cells (HSCs) in the bone marrow undergo several stages of differentiation to form monoblasts and promonocytes, which enter the circulation and differentiate into mature monocytes. During inflammation, mature monocytes migrate to renal tissue in response to the release of certain chemokines and eventually differentiate into macrophages—a process regulated by macrophage colony-stimulating factor (M-CSF, also known as CSF-1) and granulocyte‒macrophage colony stimulating factor (GM-CSF) (Lenzo et al., 2012; Sun et al., 2018).

Previous studies have employed single-cell RNA sequencing technology to investigate kidney samples derived from LN patients, thereby elucidating the intricate composition of immune cells within the kidney. These investigations have emphasized the distinct functional roles played by various subsets of macrophages in lupus nephritis. Notably, multiple macrophage subsets have been identified, including inflammatory, phagocytic, and M2-like CD16^+^ macrophages, which exhibit significant differences in their gene expression patterns and functional capabilities. Myeloid cells, particularly macrophages, hold a pivotal role in the development of lupus nephritis (LN). KRMs and MDMs play distinct pathological roles in lupus nephritis (LN). Analysis of the cell fate trajectories of KRMs and MDMs in the MRL/lpr mouse model revealed a disease-associated transcriptional state, underscoring their unique functions in the pathological process. Notably, the MDMs exhibits a pronounced induction of FcγR response genes, potentially exacerbating the autoimmune response by presenting immune complex (IC) opsonin antigens. Conversely, the LN-associated KRMs demonstrates limited immune complex uptake, yet coordinates leukocyte recruitment to inflammatory sites through the expression of monocyte chemokines, thereby driving the progression of LN. Furthermore, KRMs produces niche factors essential for B cell tissue, implying a role in lymphoid aggregates that support autoantibody production, further contributing to the development of autoimmune responses in LN (Richoz et al., 2022; Arazi et al., 2019).

Another type of cell has to be mentioned: patrolling monocytes, also known as non-classical monocytes, play an important role in promoting inflammatory activities in early LN. In the basal state, patrolling monocytes slowly crawl across the vascular endothelium using CX3CR1 and β2 integrins to act as an immune monitor of endothelial cells and surrounding tissues (Auffray et al., 2007). Once stimulated, they exude and invade the surrounding tissues rapidly, producing TNF-α and initiating the classical macrophage differentiation process (Auffray et al., 2007). Patrolling monocytes have recently been found to be present in large numbers in the glomeruli of LN patients and mice, moreover, genetic suppression of patrolling monocytes ameliorated glomerulonephritis (Kuriakose et al., 2019), suggesting a pathogenic role for patrolling monocytes in LN. The immobilized immune complex recruits patrolling monocytes directly by binding to CD16, inducing production of the neutrophil chemotactic factors CXCL2 and TNF-α (Olaru et al., 2018). Subsequently, patrolling monocytes participate in the development of renal inflammation through a series of processes including recruitment of neutrophils, upregulation of IL-6 expression, and differentiation into macrophages (Kuriakose et al., 2019; Finsterbusch et al., 2016; Nomura et al., 2022).

Macrophages are known to be highly plastic and can polarize into different phenotypes in response to various environmental stimuli. Two macrophage phenotypes were initially reported: M1 (proinflammatory or classically activated) macrophages and M2 (anti-inflammatory or alternatively activated) macrophages (Viola et al., 2019). M1 macrophages, which are generally induced by lipopolysaccharide (LPS), TNF-α and INF-γ, participate in the clearance of pathogens by increasing the level of reactive oxygen species (ROS) via nicotinamide adenine dinucleotide phosphate (NADPH) oxidase upregulation and mitochondrial damage (Kim et al., 2017; West et al., 2011). Furthermore, M1 macrophages can release high levels of proinflammatory cytokines, such as TNF-α, IL-1β, and IL-6, and therefore drive inflammatory activities (Shapouri-Moghaddam et al., 2018). In addition, the enhanced ability of antigen presentation to T cells enables M1 macrophages to activate the adaptive immune system (Arora et al., 2018). In contrast, M2 macrophages are generally anti-inflammatory and play a role in tissue repair (Wynn and Vannella, 2016; Oishi and Manabe, 2018). Fibrosis occurs through the overactivation of M2 macrophages, which function in a paracrine manner or differentiate into myofibroblast-like cells via a process called the macrophage-to-myofibroblast transition (MMT) (Fu et al., 2022; Meng et al., 2016; Luo et al., 2023). On the basis of their phenotype and function, M2 macrophages can be further categorized into four types: M2a, M2b, M2c, and M2d (Arora et al., 2018). Among them, M2a macrophages, which are induced by IL-4 and IL-13, express high levels of CD206 and are involved in tissue repair and fibrosis by producing a series of cytokines, including TGF-α and fibronectin (Toki et al., 2014; Wang et al., 2019). M2b macrophages, termed regulatory macrophages, are induced by immune complexes in combination with toll-like receptor (TLR) agonists or IL-1R agonists, and they increase the expression of the anti-inflammatory cytokine IL-10 after activation (Anderson and Mosser, 2002). M2c macrophages, which are activated by glucocorticoids, TGF-β, or IL-10, effectively inhibit inflammation and resolve tissue repair by releasing substantial amounts of IL-10 (Zizzo et al., 2012; Sapudom et al., 2021; Tang et al., 2017); however, they might contribute to fibrosis via TGF-β production (Ikezumi et al., 2015). In addition, M2c macrophages efficiently clear apoptotic cells by expressing Mer tyrosine kinase (MerTK) (Zizzo et al., 2012). M2d macrophages are induced by TLR agonists synergized with adenosine A2A receptor agonists, and they promote angiogenesis by upregulating the expression of vascular endothelial growth factor (VEGF) (Pinhal-Enfield et al., 2003). Notably, the phenotypes of macrophages are not determined by their origin but rather by the microenvironment in which they are located (Lavin et al., 2014).

Lupus flares are associated with a imbalanced ratio of M1 to M2 macrophages (Funes et al., 2018). M1 macrophages are closely associated with active SLE (Labonte et al., 2018), and polarization of M2 macrophages was observed in situations where LN is alleviated (Zhang et al., 2019). Single-cell sequencing analysis of immune cells in the kidneys of patients with LN revealed a transitional trajectory of inflammatory M1 monocytes to M2 macrophages, in which genes encoding proinflammatory cytokines were progressively downregulated, which may suggest that macrophages shift from a proinflammatory phenotype to an alternatively activated phenotype during the process of LN (Arazi et al., 2019). M1 macrophages are associated with the release of some proinflammatory mediators, including IL-1β, IL-6, TNF-α, PGE2, and ROS (Jing et al., 2020). The proinflammatory mediators expressed by macrophages are involved in tissue injury as well as the recruitment of effector cells that cause further damage to the kidney. Moreover, accumulating evidence shows that M2 macrophages also play an important role in the pathogenesis of LN. During the late stages of inflammation, M2 macrophages contribute to inflammation resolution and tissue repair. The deterioration of mice with SLE after the injection of clodronate, which selectively depletes macrophages, was ameliorated by the adoptive transplantation of M2 macrophages, suggesting that M2 macrophages play a protective role in SLE (Li F. et al., 2015).

**FIGURE 1:**
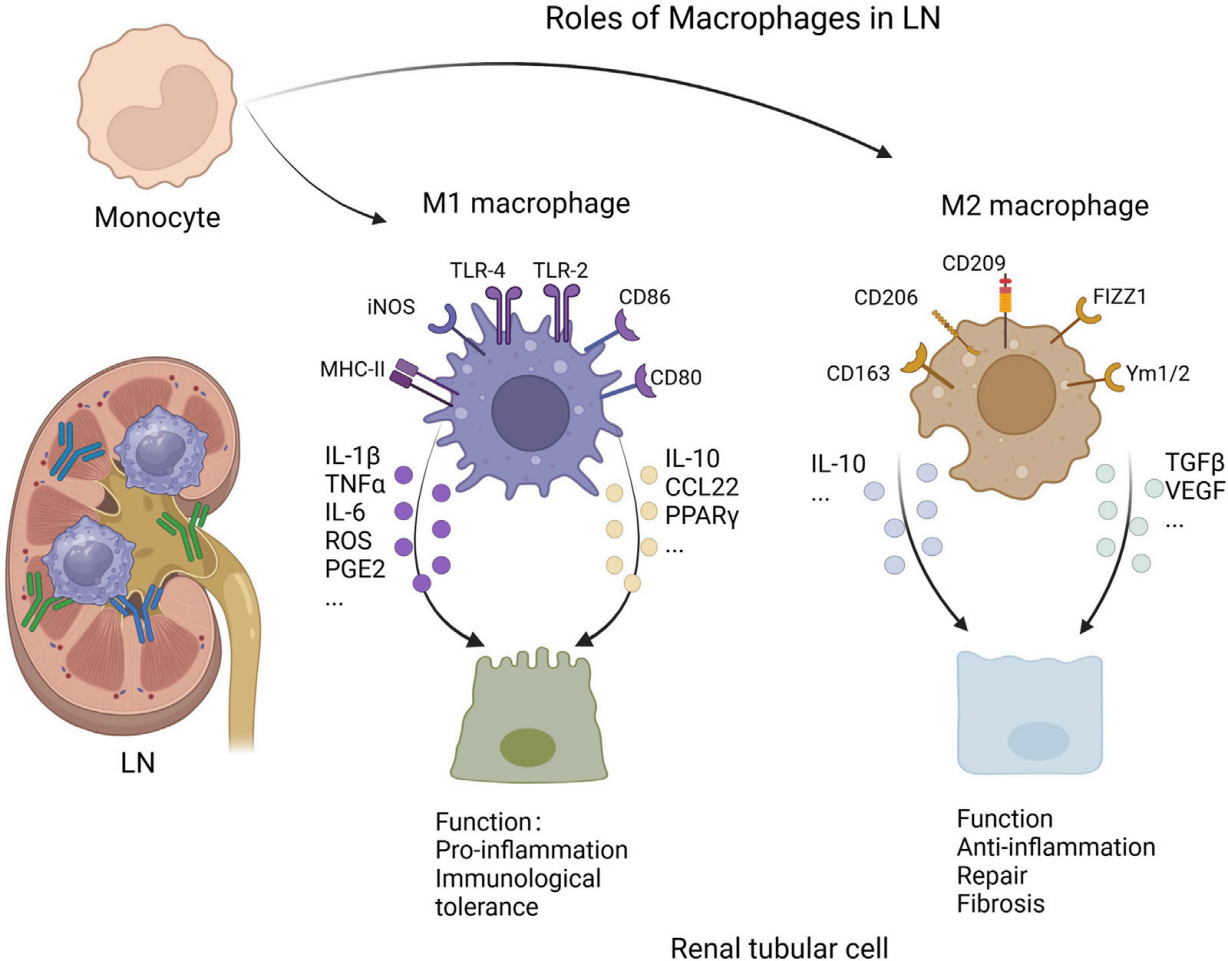
Roles of macrophages in lupus nephritis (Created with bioRender.com).

Macrophages are polarized via several different pathways in LN. As a type of extracellular vesicle, microparticles (MPs) were recently found to be a source of circulating autoantigens in SLE (Nielsen et al., 2012). Burbano et al. (2019) revealed that microparticle‐associated immune complexes (MP-ICs) induce macrophage polarization toward the M1 type, which promotes the activation of T cells and B cells in SLE. In addition, sphingosine-1–phosphate (S1P) is a bioactive lipid whose levels are increased in patients with LN (Patyna et al., 2019). Tian et al. (2023) reported that the binding of S1P to its receptor S1PR1 expressed by macrophages (Müller et al., 2017) induces the polarization of M1 macrophages through the activation of the NLRP3 inflammasome in LN. Moreover, endoplasmic reticulum stress (ERS) is involved in the polarization of macrophages. Kim et al. (2015) revealed that ERS increases the expression of proinflammatory cytokines (such as TNF-α and IL-1 β) in macrophages, suggesting a tendency toward the M1 phenotype. Furthermore, metabolic reprogramming, which is dependent on mTOR and hypoxia-inducible factor (HIF)-1α, was found to contribute to the polarization of macrophages in LN. The IgG immune complex binds to the FcγR expressed by macrophages, resulting in increased glycolysis, which induces macrophage polarization to the M1 phenotype (Jing et al., 2020). Finally, high-mobility group protein B1 (HMGB1) was found to contribute to the polarization of M1 macrophages via the TLR2/4 and NF-κB signaling pathways in patients with SLE (Schaper et al., 2016; Wang et al., 2020). In general, LN can be ameliorated by promoting macrophage polarization toward the M2 phenotype. For example, the TLR2/1 agonist PAM3CSK4 (PAM3) was found to induce the polarization of M2 macrophages, which further reduces the levels of autoantibodies and proteinuria and prolongs survival of lupus model mice (Horuluoglu et al., 2019). Thus, the progression of LN can be manipulated by regulating macrophage polarization (Figure 1).

### 2.1 Macrophages and immune tolerance in LN

As we know, macrophages are essential for immunological tolerance, and the impairment of immunological tolerance is closely related to the generation of autoantibodies to nuclear antigens. In this regard, macrophages in the splenic marginal zone (MZ) are of quite importance. MZ is defined as the transition zone between the red pulp and white pulp of the spleen, with two types of resident macrophages inside- marginal zone macrophages (MZMs) and marginal metallophilic macrophages (MMMs). In detail, MZMs highly express the C-type lectin SIGN-related 1 (SIGNR1) and the scavenger receptor MARCO, while MMMs highly express CD169 (Fujiyama et al., 2019). These macrophages induce immune tolerance by phagocytosis of apoptotic cells (McGaha et al., 2011; Ravishankar et al., 2014).

The tolerogenic function of macrophages in MZ was first described by Miyake et al. (2007), who established human diphtheria toxin receptor (DTR) transgenic mice and revealed that macrophages in MZ are specifically ablated upon injection with diphtheria toxin. They reported that the injection of apoptotic cells failed to induce tolerance in DTR mice and that the clearance of injected apoptotic cells was delayed. Further analysis revealed that macrophages in MZ modulate the selective phagocytosis of apoptotic cells by CD8α+ dendritic cells, which are responsible for immunological tolerance to autoantigens (Miyake et al., 2007). Shinde et al. (2018) revealed that DNA from apoptotic cells activates Aryl hydrocarbon receptor (AhR) in macrophages through TLR9, resulting in the production of IL-10, which is crucial for the inhibition of T-cell responses to autoantigens derived from apoptotic cells. Moreover, the chemokine CCL22 is involved in the mechanisms by which macrophages mediate immunological tolerance. CCL22 induced by apoptotic cells in the MMMs stimulates the accumulation and activation of regulatory T cells and dendritic cells, thereby resulting in the induction of the suppression of apoptotic cell antigens (Ravishankar et al., 2014). Peroxisome proliferator-activated receptor γ (PPARγ) and retinoid X receptor α (RXRα) may also be involved in the clearance of apoptotic cells by macrophages. Roszer et al. (2011) found that mice lacking macrophage expression of PPARγ or RXRα exhibit impaired clearance of apoptotic cells and high levels of autoantibodies to nuclear antigens, which are closely associated with the pathogenesis of LN.

Several studies have demonstrated a reduction in MZMs and the defective clearance of apoptotic debris by MZMs in mice that develop a lupus-like autoimmune disease. For example, the number of MZMs in BXD2 mice is significantly lower than that in C57BL/6 mice, and injected apoptotic cells are cleared more slowly in BXD2 mice than C57BL/6 mice (Li et al., 2013). RT‒PCR analysis revealed that the gene expression of tolerogenic cytokines (such as TGF-β and IL-10) by MZMs was reduced in BXD2 mice (Li et al., 2013), which may be partly responsible for the defective function of MZMs. Erythropoietin (EPO) receptors expressed by macrophages has recently been found to be correlated with the phagocytosis of apoptotic cells. Mechanically, sphingosine 1-phosphate (S1P) released by dying cells activates macrophage EPO signaling, resulting in increased expression of PPARγ, which is essential for the clearance of apoptotic cells (Luo et al., 2016). However, decreases in the levels of EPO and the EPO receptor have been observed in a pristane-induced murine lupus model (Luo et al., 2016).

### 2.2 The relationship between macrophages and renal tissue

#### 2.2.1 Macrophages and glomerular endothelial cells

The impairment of glomerular endothelial cells (ECs) is related to the development of proteinuria in LN (Nawata et al., 2018). EC proliferation, widening of the subendothelial space, and abundant infiltration of CD68^+^ (a panmacrophage marker) macrophages in the capillary lumina and subendothelial areas were observed in LN patients with endocapillary hypercellularity (EH) (Arai et al., 2021), suggesting that macrophages are involved in EC injury. In addition to secreting TNF-αthat increases the expression of heparinase by glomerular ECs, macrophages also release cathepsin L. Both heparinase and cathepsin L cause damage to the EC glycocalyx (Boels et al., 2017). The degradation of the glycocalyx further contributes to inflammatory cell extravasation and vascular endothelial injury (Yung and Chan, 2023).

Macrophages cause damage to ECs, and ECs play a role in macrophage infiltration. Zoshima et al. (2022) induced EH in mice via antibodies derived from MRL/lpr mice and analyzed the expression of chemokines and chemokine receptors. They reported that CD68^+^ macrophages, which highly express CCR2 and CX3CR1, are among the major inflammatory cells involved in EH. Moreover, ECs were found to express high levels of CCL2 and CX3CL1, suggesting that ECs are involved in the recruitment of macrophages in LN.

#### 2.2.2 Macrophages and mesangial cells (MCs)

MCs exhibit excessive cell proliferation and increased extracellular matrix secretion in LN, leading to glomerular fibrosis and sclerosis (Tsai et al., 2023). In addition, MCs are important sources of inflammatory mediators involved in the progression of LN. During the early stages of LN, anti-dsDNA antibodies bind to MCs via Annexin II (Yung et al., 2010), resulting in increased release of cytokines, chemokines and fibrotic factors (Nowling, 2022).

Proinflammatory cytokines (e.g., IL-1 and IL-6) expressed by classically activated macrophages induce MC proliferation and extracellular matrix accumulation (Ikezumi et al., 2003). Moreover, MCs play a dual role in the polarization of macrophages in LN. Liao et al. (2018) proposed that MCs activated by IFN-γ induce macrophage polarization toward the M1 phenotype in the acute nephritic phase, whereas during the chronic phase of nephritis, MCs enhance M2 polarization in the presence of growth factors to resolve inflammation and promote tissue repair.

Macrophages and MCs interact with each other through the cytokines they secrete. Sung and Fu (2020) analyzed cytokine signaling in the kidneys of lupus-prone NZM2328 mice via confocal microscopy and revealed that MCs not only are major contributors to IL-6 production in the glomerulus but also release the chemokine M-CSF, which induces the recruitment of macrophages in the glomerulus. In the initial stage of LN, deposited immune complexes promote mesangial cell activation through Fc receptors and complement. IL-6, produced by activated mesangial cells, cooperates with other cytokines to increase levels of chemokine and adhesion molecules, which lead to intraglomerular recruitment of macrophages. Subsequently, TNF-α released by macrophages induces IL-6 production in MCs, and all of these cytokines play crucial roles in events such as extracellular matrix and cellular proliferation in renal tissue (Sung and Fu, 2020).

#### 2.2.3 Macrophages and podocytes

Podocytes are highly differentiated epithelial cells. They extend many tiny processes that overlap each other around the outside of the glomerular basement membrane, forming part of the glomerular filtration barrier. The space between these processes, known as the split diaphragm, is composed mainly of nephrin, podocin, and synaptopodin, which play important roles in maintaining the structure and function of podocytes. Podocyte injury is strongly associated with the development of proteinuria in LN (Wang et al., 2014).

Podocyte injury was detected after coculture with macrophages, as evidenced by decreased synaptopodin expression and increased apoptosis (Zhang et al., 2019). Furthermore, TNF-α and IL-1β released by macrophages reduce nephrin expression in podocytes by inhibiting the promoter of nephrin (Takano et al., 2007). Moreover, TGF-β1 secreted by M2 macrophages may upregulate the expression of Snail1, matrix metalloproteinase (MMP)-7, and MMP-9 via Wnt/β-catenin signaling and downregulate the expression of nephrin, leading to podocyte mesenchymal transition, which leads to podocyte dysfunction (Wang et al., 2011; Li et al., 2008). Recently, Zuo et al. (2021) proposed that MMP-10 can cause damage to podocytes by degrading ZO-1, a podocyte tight junction protein, suggesting that classically activated macrophages may promote podocyte injury via the release of MMP-10 (Roch et al., 2014).

#### 2.2.4 Macrophages and parietal epithelial cells

Interactions between parietal epithelial cells and macrophages are rarely discussed. There is evidence that osteopontin expressed by parietal epithelial cells contributes to macrophage infiltration into the kidney (Hartner et al., 2001).

#### 2.2.5 Macrophages and the renal tubulointerstitium

Previously, researchers have focused mainly on glomerular lesions but not tubulointerstitial lesions in LN. However, alterations in tubulointerstitium are receiving increasing attention recently. Renal tubulointerstitial lesions are an independent predictor of prognosis in LN (Obrișcă et al., 2018; Gomes et al., 2021). Moreover, macrophages are abundant in the renal tubulointerstitium of patients with LN (Li et al., 2017), and their number is positively correlated with tubular interstitial inflammation and chronicity indices (Chen J. et al., 2022). In the initial phases of lupus nephritis, macrophages primarily serve a pivotal function in exacerbating inflammatory responses and presenting antigens. In the subsequent phases of inflammatory processes or injuries, macrophages play a pivotal role in facilitating tissue repair through the secretion of growth factors and extracellular matrix remodeling agents, which subsequently contributes to the development of renal tubulointerstitial fibrosis. In chronic inflammation, macrophages can form lymphoid aggregates. Lymphoid aggregates are local immune response centers at the site of inflammation, and are formed by immune cells, including macrophages and lymphocytes. These structures aid in the recruitment and activation of immune cells, maintaining local immune responses. On the one hand, lymphoid aggregates help to localize the immune response to specific areas, preventing the uncontrolled spread of inflammatory reactions. On the other hand, these aggregates provide a platform that promotes interactions between immune cells, including antigen presentation, T cell activation, and the establishment of immune tolerance. Furthermore, immune cells within lymphoid aggregates can not only modulate the intensity and duration of inflammatory responses, but also participating in tissue repair and regeneration processes. The formation of macrophages and lymphoid aggregates has a significant impact on the progression of diseases and the severity of kidney damage (Richoz et al., 2022; Jamaly et al., 2021; Lech and Anders, 2013; Khanal et al., 2023). In addition, macrophage depletion by the administration of diphtheria toxin has been shown to ameliorate tubular injury and interstitial fibrosis in mice with crescentic glomerulonephritis, suggesting that macrophages play an important role in the generation of renal tubulointerstitial lesions (Duffield et al., 2005).

The effects of macrophages on tubular injury are closely related to NF-κB activation. Research has shown that activated NLRP3 in renal tissue-resident macrophages upregulates the expression of IL-33, which promotes inflammatory activities in renal tubular epithelial cells (TECs) via the IL33/ST2/NF-κB pathway. Inhibition of these processes reduces tubular injury and thereby alleviates LN (Ma et al., 2023). Moreover, activation of the NF-κB signaling pathway in macrophages is associated with the release of proinflammatory cytokines (such as IL-6, IL-1 and TNF-α) (Mussbacher et al., 2023). Immunoreactive CD11c+ macrophages derived from circulating monocytes have been identified in the urine of patients with LN. Kim et al. (2020a) reported that CD11c+ macrophages expressing the chemokine receptor CXCR3 are recruited to the renal tubulointerstitium via the chemokine IP-10 produced by TECs. Activated CD11c+ macrophages release high levels of IL-6, which increases fibronectin expression and promotes TEC detachment and apoptosis (Kim et al., 2020a). Among them, fibronectin can be deposited in the tubulointerstitium, inducing the development of interstitial fibrosis (Eddy, 2000), whereas the apoptosis of renal tubular epithelial cells is closely related to tubular atrophy (Havasi and Borkan, 2011). In addition, macrophage exosomes enriched with miR-155 were identified by Zhang Z. et al. (2022). These exosomes can promote inflammatory activities in tubular epithelial cells by downregulating the expression of SOCS-1, a negative regulator of NF-κB, and the inhibition of miR-155 ameliorates renal tubular injury *in vivo* and *in vitro* (Zhang Z. et al., 2022).

Analysis of animal models revealed that M2 macrophages are crucial for the fibrotic phase of kidney disease (Han et al., 2013; Kushiyama et al., 2011). Macrophages produce a series of cytokines, such as IL-1, matrix metalloproteinases, and platelet-derived growth factor, which promote the proliferation and activation of fibroblasts (Tang et al., 2019). Myofibroblasts are activated fibroblasts that produce pathogenic collagen, contributing to the development of tubulointerstitial fibrosis (Klingberg et al., 2013). Interestingly, M2 macrophages can also differentiate into myofibroblasts via a process known as the macrophage–myofibroblast transition (MMT), which is regulated by TGF-β/Smad3 signaling, resulting in the production of collagen (Wang et al., 2016).

Renal tubular epithelial cells are not only targets but also drivers of inflammatory activities in LN. They promote macrophage proliferation and survival through the expression of CSF-1 and IL-34 (Wada et al., 2019) and macrophage recruitment through chemokines such as CCL2 and CX3CL1 (Jia et al., 2022a; Jia et al., 2022b; Kassianos et al., 2015). In addition, exosomes produced by TECs were recently shown to be associated with the activation of inflammatory macrophages (Lv et al., 2020; Lv et al., 2018; Li et al., 2019). Once internalized by macrophages, these exosomes, which can be enriched either with microRNAs or CCL2, promote the polarization and migration of M1 macrophages.

## 3 Macrophages and their products serve as biomarkers in LN

The traditional indicators used to test for LN include serum creatinine, urinary protein, anti-dsDNA antibodies and complement C3/4. However, these indicators cannot directly reflect LN flares or distinguish between active and chronic disease. Renal biopsy is the gold standard for the diagnosis of LN, and repeat renal biopsy is recommended for monitoring disease status and guiding the treatment of LN patients (Pakozdi et al., 2018; Marinaki et al., 2020; Lledó-Ibáñez et al., 2022). Nevertheless, renal biopsy is an invasive procedure that cannot be performed anytime or anywhere, which limits its clinical application. Therefore, safe, noninvasive and simple methods are needed for the diagnosis and monitoring of LN.

Intraglomerular CD68^+^ macrophages can be used as biomarkers for EH to reduce variation due to observer subjectivity. Bos et al. (2022) reported that the number of CD68^+^ macrophages in the glomerulus was significantly correlated with EH in LN. They reported that when 7 or more CD68^+^ macrophages were present in a glomerulus, the diagnostic sensitivity and specificity of intraglomerular CD68^+^ macrophages for EH were 88% and 67%, respectively.

Urinary CD11c+ macrophages derived from circulating monocytes are abundant in the urine of patients with active proliferative LN and are significantly associated with the serum anti-dsDNA antibody titer, renal tubular atrophy and interstitial fibrosis (Kim et al., 2020b). More importantly, the number of urinary CD11c+ macrophages is significantly lower in patients who achieved a complete or partial renal response than in those who did not achieve a renal response after 6 months of treatment (Kim et al., 2020b). These results suggest that the number of urinary CD11c+ macrophages may serve as a biomarker reflecting the clinical and pathological characteristics, as well as the treatment response, of patients with LN.

Soluble CD163 is the most discussed macrophage product. It is derived from the cleavage of the CD163 macrophage receptor (Møller et al., 2010) and can be detected in the urine of LN patients (Endo et al., 2016). Compared with those in SLE patients without LN, patients with other glomerular diseases, and healthy controls, the levels of urinary soluble CD163 in patients with active LN were shown to be significantly greater (Mejia-Vilet et al., 2020; Zhang et al., 2020), suggesting that urinary soluble CD163 may serve as a biomarker of LN. Urinary CD163 begins to rise 6 months before the onset of LN and is not only closely related to the Systemic Lupus Erythematosus Disease Activity Index (SLEDAI) and renal histopathological changes but also reflects the effectiveness of treatment (Mejia-Vilet et al., 2020; Zhang et al., 2020; Gupta et al., 2021), making it an excellent and non-invasive marker during LN follow-up. In contrast, the percentage of CD163+ macrophages in the renal tubulointerstitial compartment is positively correlated with the chronicity index in LN (Allam et al., 2020).

## 4 Targeting macrophages for LN intervention

Current treatments for LN tend to be glucocorticoids and immunosuppressants (Table 1), which not only exhibit variable efficacy but also lead to potentially serious adverse events. Given the role of macrophages in the pathogenesis of LN and the promising results of macrophage-associated targeted therapies in a variety of animal models, treatments targeting macrophages, including the promotion of phagocytosis by macrophages, the inhibition of monocyte/macrophage recruitment, the modulation of macrophage polarization and the inhibition of macrophage cytokine production, may be potential interventions for LN. However, most of the experiments evaluating these treatments have been performed in animals and need to be confirmed by further clinical trials.

**TABLE 1:** Intervention LN compounds and targets summary.

Reagents	Targets	Mechanisms
ARA290 (Huang et al., 2018)	EPO derivative	Inhibit the activation of M1 macrophages and the production of inflammatory cytokines (such as IL-6 and TNF-α)
Mizoribine (Tanaka et al., 2010)	Inhibit purine synthesis	Inhibit infiltration of macrophages in glomeruli
Disulfiram (Terashima et al., 2020; Toda et al., 2009; Toda et al., 2022)	Inhibit FRONUT	Negatively regulate monocyte/macrophage migration Reduce production of cytokine and chemokines (for example, TNF-α and CCL2) in macrophages
GW2580 (Chalmers et al., 2015; Chalmers et al., 2017)	Selective inhibitor of CSF-1 receptor kinase	Deplete macrophages
(5R)-5-hydroxytriptolide (Zhang et al., 2017)	Inhibit the expression of chemokines	Reduce the infiltration of immune cells (like macrophages, T cells, and neutrophil cells)
PI3Kδ inhibitors (Okkenhaug and Fruman, 2010)	Inhibit PI3Kδ	Attenuate the ability of macrophages to cross the glomerular basement membrane
glycolysis inhibitors (Jing et al., 2020)	Inhibit Glyolysis	Manipulate macrophage metabolism
mivebresib (Bignon et al., 2014)	Inhibit BRD4	Inhibit M1 polarization of macrophages
Serp-1 (He et al., 2023; Zhuang et al., 2021)	serpin encoded by myxomavirus	Inhibit M1 polarization of macrophages
X receptor-alpha (He et al., 2023; Zhuang et al., 2021)	X receptor-alpha	Promote the polarization of M2 macrophages
supplementation of serum amyloid p component (SAP) (Zhang et al., 2011)	Macrophages	Shift macrophages from a pro-inflammatory M2b to an anti-inflammatory M2a phenotype
mannose-binding lectin (MBL) (Cai et al., 2013)	inhibit the MAPK and NF-κB signaling pathways	Reduce M2b polarization
sedum sarmentosum bunge extract (Lu et al., 2019)	Inhibit M1 macrophage polarization	Inhibit M1 macrophage polarization
PAM3CSK4 (Horuluoglu et al., 2019)	TLR2/1 agonist	Promote M2 macrophage polarization
Mesenchymal stem cells (MCs) (Zhang et al., 2019)	Macrophages	Promote the anti-inflammatory phenotype of macrophages
Cf-02 (Yang et al., 2021)	Anti-inflammatory	Reduce the infiltration of inflammatory cells

Since EPO signaling plays an important role in the clearance of apoptotic cells by macrophages, interference with EPO signaling may be a promising approach for LN intervention. Using a lupus-prone mouse model, Luo et al. (2016) found that recombinant human EPO or PPARγ agonists reduced autoantibody production and improved renal function. However, the application of EPO agonists may increase the risk of several adverse effects, including hypertension, thromboembolism, and cardiovascular events (Sonia et al., 2023). Hence, an EPO derivative named ARA290 that possesses the anti-inflammatory and cytoprotective properties of EPO but has no effect on the hematopoietic system was developed (Brines et al., 2008). ARA290 has been reported to significantly promote the phagocytosis of apoptotic cells by macrophages in lupus-prone mice, in addition to inhibiting the activation of M1 macrophages and the production of inflammatory cytokines (such as IL-6 and TNF-α), all of which contribute to the alleviation of renal inflammation in LN (Huang et al., 2018).

Moreover, the inhibition of monocyte/macrophage recruitment alleviates renal inflammation by directly reducing the number of inflammatory macrophages. Mizoribine is a purine synthesis inhibitor that blocks macrophage infiltration by decreasing OPN expression, thereby ameliorating fibrosis in animal models (Takahashi et al., 2009; Sato et al., 2001). In patients with LN, mizoribine significantly inhibits infiltration of macrophages in the glomeruli and reduces the chronicity index, with no serious adverse events observed (Tanaka et al., 2010). However, the sample size of that trial was insufficient, and further large-scale studies are needed. As an alcohol withdrawal drug, disulfiram has recently been reported to negatively regulate monocyte/macrophage migration by inhibiting FRONUT, a cytoplasmic protein that promotes chemotaxis by interacting with CCR2 and CCR5 (Terashima et al., 2020; Toda et al., 2009). In addition, disulfiram reduces the production of cytokines and chemokines (for example, TNF-α and CCL2) in macrophages and thereby alleviates renal pathological injury and proteinuria (Toda et al., 2022). In addition, treatment of lupus-prone mice with GW2580, a selective inhibitor of CSF-1 receptor kinase, depletes macrophages in the glomeruli and significantly reduces albuminuria and the levels of serum creatinine, as well as renal tissue damage (Chalmers et al., 2015; Chalmers et al., 2017). Recently, (5R)-5-hydroxytriptolide was shown to reduce the infiltration of immune cells (such as macrophages, T cells, and neutrophils) in MRL/lpr mice by inhibiting the expression of chemokines. Moreover, reductions in serum creatinine levels and proteinuria, reduced renal damage, and increased life spans were also observed in mice treated with (5R)-5-hydroxytriptolide (Zhang et al., 2017). PI3Kδ, which is expressed mainly in hematopoietic cells (Vanhaesebroeck et al., 1997), regulates the differentiation and cytokine production of B and T cells (Okkenhaug and Fruman, 2010). Surprisingly, PI3Kδ inhibitors reduced the number of B cells and T cells in MRL/lpr mice while inhibiting the ability of macrophages to cross the glomerular basement membrane, which is critical for intrarenal macrophage infiltration (Suárez-Fueyo et al., 2014). PI3Kδ inhibitors are not only effective at ameliorating kidney damage and prolonging the life span of mice but also safe (Suárez-Fueyo et al., 2014), making them promising drugs for the treatment of LN. Other drugs that inhibit the infiltration of macrophages and a variety of other immune cells include CCR1 antagonists, which prolong survival in NZB/W mice by alleviating renal pathological injury and delaying fatal proteinuria (Bignon et al., 2014).

Metabolic reprogramming is a key regulator of macrophage polarization. M1 macrophages typically use glycolysis to generate energy, while M2 macrophages use oxidative phosphorylation (Galván-Peña and O’Neill, 2014). Jing et al. (2020) treated primary human renal macrophages and nephrotoxic serum-challenged mice with glycolysis inhibitors and reported that the release of the proinflammatory mediator IL-1β by macrophages was reduced; moreover, the mice presented reduced serum urea levels and IL-1β-mediated inflammatory cell recruitment to the kidney. These findings suggest that manipulating macrophage metabolism may interfere with the progression of LN. In addition, mivebresib, a BRD4 inhibitor, and Serp-1, serpin encoded by myxomavirus, have recently been shown to play roles in SLE-associated diffuse alveolar hemorrhages by inhibiting the polarization of M1 macrophages, showing promise for LN treatment (He et al., 2023; Zhuang et al., 2021). Liver X receptor-alpha achieves a similar effect by promoting the polarization of M2 macrophages (Han et al., 2018). It has also been reported that supplementation with serum amyloid p component (SAP) can alleviate LN in mice by shifting macrophages from a proinflammatory M2b phenotype to an anti-inflammatory M2a phenotype (Zhang et al., 2011). However, supplementation with mannose-binding lectin (MBL) inhibits the MAPK and NF-κB signaling pathways, which in turn reduces M2b macrophage polarization and exerts beneficial effects on the clinical and pathological parameters of LN patients (Cai et al., 2013). Other drugs that inhibit M1 macrophage polarization, including Sedum sarmentosum Bunge extract (Lu et al., 2019), and drugs that promote M2 macrophage polarization, including the TLR2/1 agonist PAM3CSK4 (Horuluoglu et al., 2019), the AhR agonist indole-3-carbinol (Mohammadi et al., 2018), the PPAR γ agonist pioglitazone (Mohammadi et al., 2017), and total peony glucosides (Liang et al., 2021), have been shown to have therapeutic effects on SLE or LN. Mesenchymal stem cells (MSCs), including bone marrow-derived MSCs (BM-MSCs), umbilical cord-derived MCs (UC-MSCs) and adipose tissue-derived MSCs (AT-MSCs), play multiple roles in LN, one of which is the induction of an anti-inflammatory phenotype in macrophages. *In vivo* and *in vitro* studies have shown that human UC-MSCs can not only reduce macrophage infiltration but also induce the polarization of anti-inflammatory macrophages to prevent podocyte injury in lupus-prone mice (Zhang et al., 2019). Deng et al. (2015) proposed that the reduced levels of macrophage CD206 and phagocytic activity in patients with SLE can be restored by human UC-MSCs, in which IL-6 plays a key regulatory role. BM-MSCs have also been reported to significantly reduce the number of macrophages in rats with chronic renal failure (Caldas et al., 2015). Interestingly, it has been suggested that MSC-derived exosomes possess immunomodulatory effects similar to those of MSCs. *In vitro* experiments demonstrated that exosomes derived from AT-MSCs could induce macrophages to differentiate into an immunosuppressive phenotype, which is related to an increase in the number of regulatory T (Treg) cells (Özdemir et al., 2019). Treg cells are protective cells that contribute to the alleviation of SLE (Scheinecker et al., 2020). Moreover, macrophages incubated with MSC-derived exosomes exhibited reduced proliferative capacity, decreased expression of LPS-induced inflammatory cytokines, and an increased percentage of M2 macrophages (Sun et al., 2022). Moreover, MRL/lpr mice injected with MSC-derived exosomes presented reduced deposition of glomerular collagen fibers and complement C3, increased levels of serum IL-10, and prolonged survival, suggesting that MSC-derived exosomes may ameliorate LN by inducing M2 macrophage polarization (Sun et al., 2022). Specifically, the mechanism by which MSC-derived exosomes induce the M2 phenotype in macrophages may be partially attributable to the downregulation of NOTCH1 expression by miR-146a-5p in exosomes (Chen X. et al., 2022). In addition, Zhang M. et al. (2022) showed that exosomes derived from BM-MSCs may promote M2 macrophage polarization via the miR-16 and miR-21 they carry, resulting in a reduction in antibodies and complement deposition in the glomerular mesangial and endocapillary regions. In addition, exosome-treated macrophages increase the expression of CCL20, which is closely related to the expansion of Treg cells (Zhang M. et al., 2022). All of these factors have been shown to contribute to the amelioration of LN.

Glucocorticoids are the most widely used drugs in the treatment of LN. As reviewed by Ehrchen et al. (2019), they act not only by reducing the expression of proinflammatory mediators (such as IL-1β, IL-6, IL-12 and TNF-α) but also by inducing the expression of anti-inflammatory mediators (Fkbp5, MKP-1, Tsc22d3, Per1, adenosine receptor 3a and formyl peptide receptor) in monocytes/macrophages. These effects are essential for ameliorating inflammation in the kidney. Cf-02, a synthetic compound, has recently shown favorable therapeutic effects in NZB/WF1 mice with acute-onset LN that induced by LPS intraperitoneal injections (Yang et al., 2021). Specifically, cf.-02 reduces the infiltration of inflammatory cells, including macrophages, and inhibits the activation of the macrophage NLRP3 inflammasome, which ultimately ameliorates renal function, proteinuria, and renal histopathology (Yang et al., 2021). Additionally, paeoniflorin (Ji et al., 2018), luteolin (Ding et al., 2023), and quercitrin (Li et al., 2016) have been found to be potent anti-inflammatory agents that reduce the production of proinflammatory cytokines by inhibiting NF-κB activation in macrophages, suggesting potential therapies for LN. Isogarcinol, a natural compound extracted from Garcinia mangostana L., has been shown to be an immunomodulatory drug with low cytotoxicity (Cen et al., 2013). Li et al. reported that isogarcinol abrogated the increased secretion of IL-1 β, IL-6 and TNF-α in macrophages induced by LPS, and decreased expression of proinflammatory cytokines was also observed in the kidneys of mice treated with isogarcinol (Li W. et al., 2015), suggesting that isogarcinol may be a candidate anti-inflammatory drug for kidney protection in LN.

## 5 Conclusion

Macrophages have various roles in lupus nephritis (LN), including inflammatory responses, tissue repair, immune tolerance, and their interaction with intrinsic cells in the kidney. Macrophages have cellular interactions with glomerular endothelial cells, mesangial cells, podocytes, and renal tubular epithelial cells to affect the progression of LN. The polarization status of macrophages is closely correlated with the pathogenesis of LN, where M1 type macrophages are associated with disease activity, while M2 type macrophages are associated with the resolution of inflammation and tissue repair. Moreover, the potential of macrophages and their products as biomarkers in the diagnosis and monitoring of LN, and therapeutic strategies targeting macrophages provide new perspectives for the treatment of LN. Macrophages with different polarization statuses participate in different stages of LN development. A comprehensive understanding of the mechanisms by which macrophages are involved in the development of LN is crucial for exploring treatments for LN, but the heterogeneity of macrophages poses numerous challenges to our research. Current therapies targeting macrophages have shown promising results in lupus animal models; however, these effects may not be observed in humans. Key issues to be addressed in future research include the identification of optimal macrophage-targeted therapeutic strategies, the evaluation of the safety and efficacy of these therapies in humans, and the development of new biomarkers to improve the diagnosis and treatment of LN.

In conclusion, macrophages play complex roles in LN, which not only participate in the pathogenesis of the disease, but may also serve as new therapeutic targets. By gaining insight into the role of macrophages in LN, we can develop more effective and safer treatments for LN patients.
